# Unilateral proptosis: a rare presentation of metastatic prostate cancer

**DOI:** 10.1093/jscr/rjad605

**Published:** 2023-11-10

**Authors:** Gaurav Sharma, Michael Wanis

**Affiliations:** Department of Urology, East Surrey Hospital, Redhill, Surrey & Sussex Healthcare NHS Trust, RH1 5RH, United Kingdom; Department of Urology, East Surrey Hospital, Redhill, Surrey & Sussex Healthcare NHS Trust, RH1 5RH, United Kingdom

**Keywords:** prostate cancer, cranial metastasis, proptosis

## Abstract

Prostate adenocarcinomas with metastatic cranial involvement are rare, with signs and symptoms overlapping with those of the primary base of the skull tumour. The diagnosis was made following a biopsy of the suspected lesions that indicated the possibility of a prostatic primary malignancy based on immunohistochemistry using prostate-specific membrane antigen and subsequently confirmed histologically. We report an unusual case of a 52-year-old male who presented with unilateral proptosis and no prior urological history. Cranial, pulmonary, and thoracolumbar spinal metastases were identified with radiological imaging. We describe the diagnostic evaluation and treatment, as well as outline the rare nature of this case of cranial metastasis of prostate cancer.

## Introduction

Prostate adenocarcinoma is the most common cancer in men, which accounts for >20% of newly diagnosed cancers [1]. Most prostate cancer (PCa) cases are diagnosed in the early stages; however, up to 5% of patients present with de novo metastases [2]. The common metastasis sites are the pelvic lymph nodes and bones. Other visceral organs, such as the liver, lungs, and adrenal glands, are rarely involved. Cranial metastasis occurs in 7% of metastatic PCa cases [3]. The occipital bone is most commonly affected in the cranium, probably due to tumour cell seeding via Batson’s vertebral venous plexus [4]. Patients who have PCa with cranial metastasis (with positive radiological evidence) can be asymptomatic or show symptoms such as bone pain, cranial nerve paresis, or a palpable mass, which is usually osteoblastic [3, 5, 6].

## Case report

**Figure 1:**
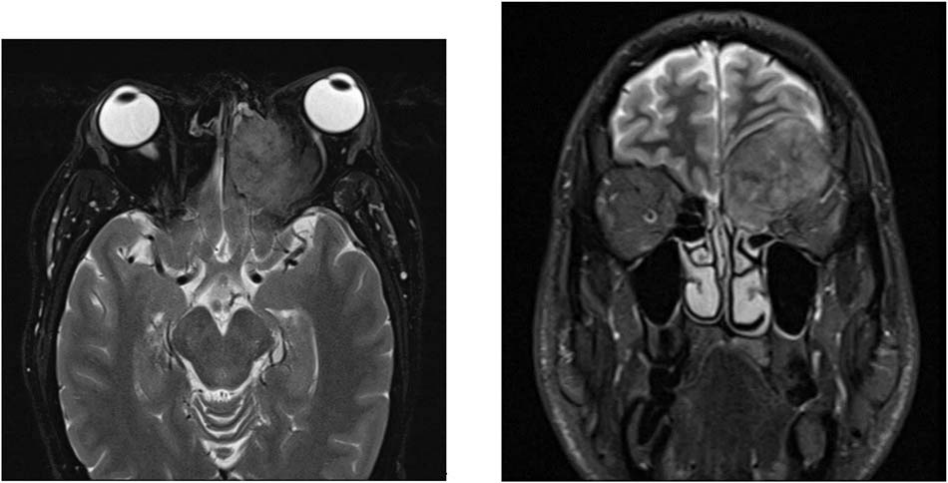
MRI with contrast shows a large left anterior cranial fossa and extra-axial extradural solid mass involving the left olfactory groove with bony erosion. There is a large component on the superior and medial extraconal orbital causing left proptosis. Direct involvement of the left frontal sinus is observed. (**a**) Axial view. (**b**) Coronal view.

**Figure 2:**
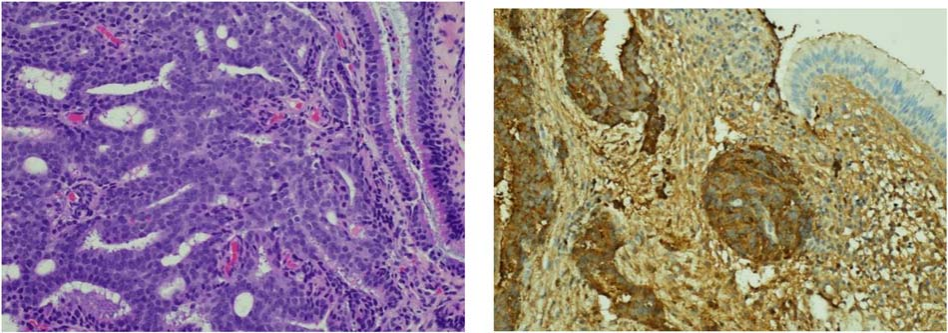
Histological findings. (**a**) Respiratory epithelium showing infiltration by a tumour comprising cribriform sheets of relatively uniform rounded tumour cells with prominent nucleoli in keeping with adenocarcinoma. Morphological features along with immune profile (keratin positive, negative for CK 7 & 20) pointed to a prostatic primary. (**b**) Positive immunohistochemistry staining with PSA marker.

A 52-year-old male initially presented with preseptal cellulitis, which was successfully treated with antibiotics. One month later, he developed protrusion of the left eye with numbness over the left eyelid and ipsilateral forehead. No diplopia or signs of optic nerve compression were observed. A computed tomography (CT) scan revealed a large mass sized 36 × 31 x 29 mm centred over the left orbital roof, damaging bone and extending superiorly into the anterior cranial fossa, inferiorly into the orbit, as well as medially into the left olfactory and left nasal fossa (Fig. 1). It was reported as a sclerotic lesion with soft tissue elements. Another 25-mm lesion in the skull base infiltrated the right side of the clivus. These findings were confirmed by magnetic resonance imaging (MRI) of the head. The findings were then discussed at the Head & Neck multidisciplinary team meeting (MDT), where, considering the possibility that these might be metastatic deposits, an MRI was recommended to further characterize the lesion. It revealed extradural involvement of the left anterior cranial fossa without any evidence of intracerebral or leptomeningeal disease. Additionally, a thorax, abdomen, and pelvis CT with contrast showed multiple aggressive sclerotic lesions in the thoracolumbar spine and multiple nodules in the upper lobe of both lungs. No invasive mass in the prostate or abdomen, or inguinal lymphadenopathy was observed. Based on these findings, the MDT meeting recommended a transnasal tissue biopsy, which revealed an adenocarcinoma (Fig. 2). Immunohistochemical results were keratin positive, largely negative for CK 7 and 20, as well as negative for other markers, suggesting a prostatic origin. Prostate-specific antigen (PSA) marker test of the tumour cells was positive, indicating the prostate as the primary site.

**Figure 3:**
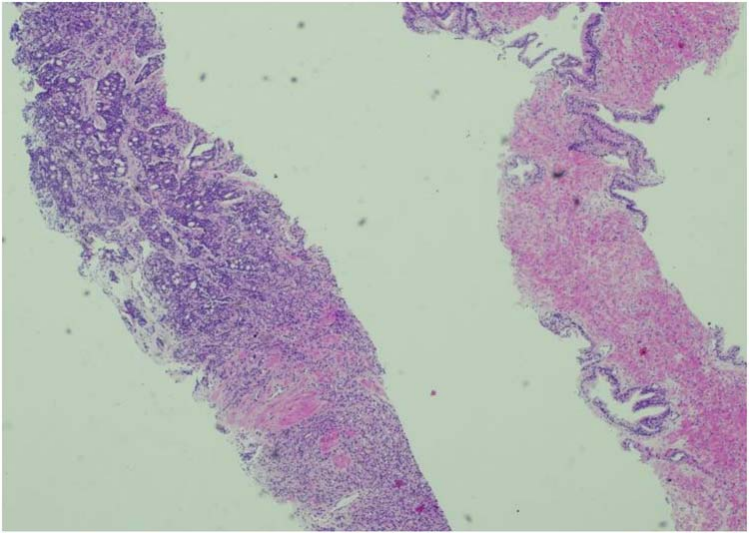
Histology of prostatic tissue showing adenocarcinoma. Gleason score 5 + 4 = 9, Gleason group 5. 5/24 cores were positive for adenocarcinoma.

**Figure 4:**
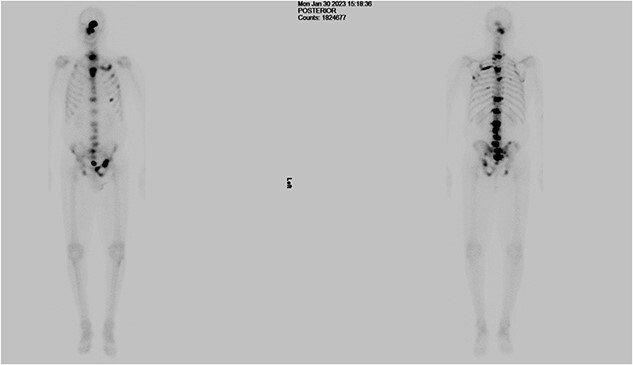
Bone scan showing multiple areas of increased tracer uptake, including the skull vault and skull base, cervical spine, thoracic spine, lumbar spine, and pelvis, revealing multiple bony metastases.

The patient was then referred to the urology department by the Head & Neck MDT. As part of his workup, a digital rectal examination (DRE), PSA test, prostate biopsy, and bone scan were recommended, and gonadotropin-releasing hormone (GnRH) antagonists were started. The DRE revealed a mildly enlarged, firm, smooth prostate with no nodularity. The PSA level was 1271 ng/dl, which decreased to 639 g/L within 1 week of the commencement of treatment. Transrectal ultrasound revealed a prostate volume of 54 cc and a local anaesthetic transperineal template biopsy of the prostate revealed prostatic adenocarcinoma with 4 out of 12 cores from the right gland with a Gleason Score of 4 + 5 and 1 out of 12 cores from the left gland with a Gleason Score of 5 + 5. The mean overall Gleason score was 5. Additional features included a cribriform pattern and perineural invasion (Fig. 3). Bone scans revealed multiple areas of increased tracer uptake, including the skull vault and skull base, cervical spine, thoracic spine, lumbar spine, and pelvis, with multiple bony metastases (Fig. 4). Therefore, the staging of the disease was T1c N0 M1b, high-risk, or Cambridge Prognostic Group 5.

Subsequently, the patient was referred to the oncology department for further treatment, where the GnRH antagonists were replaced with luteinizing hormone-releasing hormone analogues, and calcium and vitamin D were prescribed for bone protection.

## Discussion

PCa is the most commonly diagnosed cancer in men worldwide. The spread of PCa occurs via direct local invasion, perineural invasion, or the bloodstream and lymphatic system. Haematogenous spread occurs mainly in the bones. Distant visceral metastases are observed in the pelvic lymph nodes, liver, and lungs. Nearly all patients with advanced PCa exhibit clinically evident bone metastases that commonly spread to the lumbar spine, ribs, and pelvis [7]. The involvement of skull convexity is frequent, but the skull base and orbits are less commonly affected [8].

PCa metastasis to the orbit typically presents with signs of diplopia, proptosis, ophthalmoplegia, ptosis, red eye, rapidly progressive pain, or decreased visual acuity which, in up to 25% of patients, can be the initial presenting signs of a primary tumour [9]. Symptoms of metastasis to the nearby paranasal sinuses can mimic sinus infection and present as diplopia, nasal mass or obstruction, and nasal pain [10]. Metastases to the eye and orbit typically occur via haematogenous spread through the carotid and ophthalmic arteries.

Genitourinary cancers may access this route via preexisting pulmonary metastases or Batson’s plexus [9], and the clinical presentation of metastases to the orbital region depends on the structures affected. For example, pain, diplopia, and decreased visual acuity may be present if the orbital bone, soft tissue, or globe are affected. These symptoms may progress over weeks or months. Examination may reveal ptosis, proptosis, ophthalmoplegia, or red eye. Complications include uveitis, papilledema, retinal detachment, and secondary glaucoma [9].

**Figure 5:**
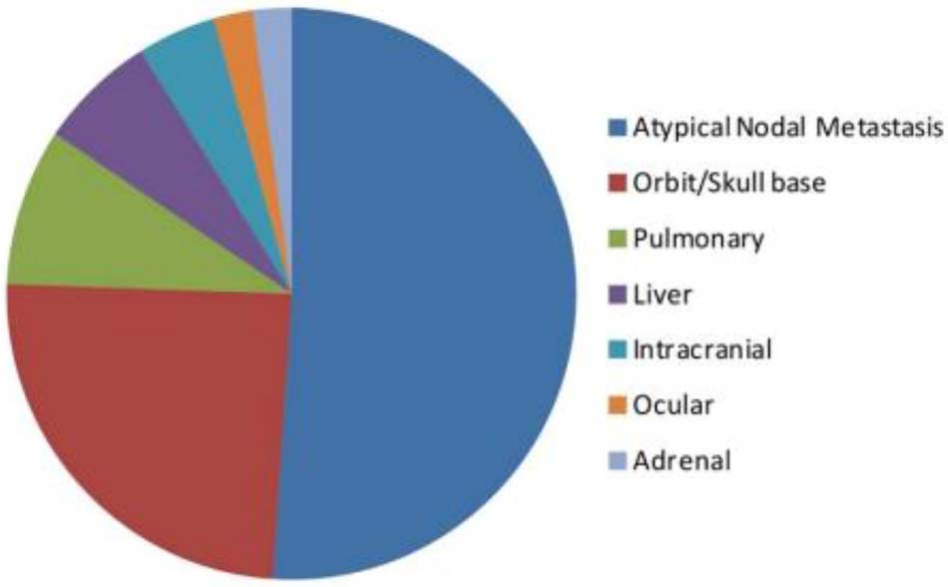
Distribution of atypical metastasis from PCa.

The orbit is the second most common site for unusual PCa metastasis, after atypical lymph node metastasis (Fig. 5) [11].

The prognosis of these patients depends on the effectiveness of hormonal treatment. Endocrine treatment and palliative external beam radiotherapy remain the standard treatment for vision problems [12]. Despite overall good early PCa detection rates, unusual presentations of the advanced metastatic disease continue to be diagnosed. Whenever signs of the base of skull tumours are observed without a known primary origin, a prostatic primary should be considered in the potential differential diagnosis.
